# Supplementary material for: Physiological Effects and Mechanisms of Chlorella vulgaris as a Biostimulant on the Growth and Drought Tolerance of Arabidopsis thaliana
Source: Plants (Basel). 2024 Oct 28;13(21):3012. doi: 10.3390/plants13213012 (PMC11548328; doi:10.3390/plants13213012)
Supplement: Supplementary file 1 [file plants-13-03012-s001.zip › plants-3226599-supplementary.pdf]

Supplementary Information

Physiological Effects and Mechanisms of *Chlorella vulgaris* as a Biostimulant on the Growth and Drought Tolerance of *Arabidopsis thaliana*

Jinyoung Moon [1], Yun Ji Park [1,2], Yeong Bin Choi [1,3], To Quyen Truong [1], Phuong Kim Huynh [1,3], Yeon Bok Kim [4] and Sang Min Kim [1,3,*]

[1] Smart Farm Research Center, Korea Institute of Science and Technology (KIST), Gangneung Institute of
Natural Products, Gangneung 25451, Republic of Korea; jy77mun@kist.re.kr (J.M.); yunji0825@changwon.ac.kr (Y.J.P.); choiyeongbin@kist.re.kr (Y.B.C.); ttquyen_vn@kist.re.kr (T.Q.T.); 623024@kist.re.kr (P.K.H.)

[2] Gyeongnam Bio and Anti-Aging Core Facility Center, Changwon National University, Changwon 51140, Republic of Korea

[3] Natural Product Applied Science, KIST School, University of Science and Technology, Seoul 02792, Republic of Korea

[4] Department of Medicinal and Industrial Crops, Korea National University of Agriculture & Fisheries,
Jeonju 54874, Republic of Korea; yeondarabok@korea.kr

* Correspondence: kimsm@kist.re.kr; Tel.: +82-33-650-3640; Fax: +82-33-650-3679

Table S1. Gene-specific primer sequences used in RT-qPCR

Gene	Forward Primer Sequence	Reverse Primer Sequences
PP2A	TATCGGATGACGATTCTTCGTGCAG	GCTTGGTCGACTATCGGAATGAGAG
BES1	CACGGGATACGTGTGAACTG	GAACAGCGTTCGCTACATCA
RD26	AGAGCATAGCACGAATGGGT	TTCTGCTGCCGATTCACATG
SMXL6	AAAGCTGAGTTCCCGGATCA	TGAACGTTGCACCTTCACTG
IAA5	TGAAATGTGAACCGGCGAAA	CCAGCAAGCATCCAATCTCC
IAA6	GAGGGTGCTCTCGGATATGA	TATGCCAAGACAGCCGAAGA
IAA19	GCCACCGGTTTGTTCTTACC	CGAGCATCCAGTCTCCATCT
WRKY63	CGTCAACCACACCATGAGTC	GTCTTGGATCATCTGCACCC
MYB28	CACCGATTCAAGGCAGTTCC	AACGATGATGGGGAGAAGGG
MYB29	CTAGGGCTTCTTCCATGGGA	TCAGCTTCTTCCCCTTCGTT
WRKY18	CGAGCAGACCGATATACCGA	AACTGTCAAGCTTGTGTCCG
WRKY40	GCTTCTGACACTACCCTCGT	GGTGTTGAAGCTGAACCACC
WRKY60	TGCTGCTGAGAAGTCTGACA	CTCAACTGGTTCAAGCCCAC
AREB1	CGCCATTGTCATCAGAAGGG	ATCCTTGCCTGCTTTCGTTG
ABF3	CAACGGTGGGGACTAGTCTT	ACCAATGTTGTTACCACCGC
DREB2A	TTGGCTGAGCGAGTTTGAAC	GACTCTCGGGCCTGTATGAA

Table S2. Relative expression level of drought stress responsive genes in *A. thaliana* treated with CB, CFS, and CS by RT-qPCR

**Genes**	**Treatments**		
	**CB**	**CFS**	**CS**
BES1	1.637 ± 0.451	1.965 ± 0.329	1.419 ± 0.391
RD26	0.559 ± 0.029	0.424 ± 0.073	0.965 ± 0.224
SMXL6	1.938 ± 0.993	2.753 ± 1.016	2.483 ± 1.113
IAA5	1.117 ± 0.016	24.250 ± 2.337	1.007 ± 0.020
IAA6	4.021 ± 0.734	5.266 ± 1.701	3.982 ± 0.574
IAA19	2.146 ± 0.799	1.078 ± 0.227	0.779 ± 0.149
WRKY63	1.031 ± 0.149	0.497 ± 0.113	3.669 ± 0.393
MYB28	2.899 ± 1.200	4.358 ± 0.528	3.558 ± 0.348
MYB29	3.369 ± 1.240	3.662 ± 0.503	4.999 ± 0.870
WRKY18	0.630 ± 0.130	0.129 ± 0.027	0.072 ± 0.023
WRKY40	1.723 ± 1.133	0.624 ± 0.200	0.179 ± 0.049
WRKY60	1.741 ± 0.150	2.005 ± 0.506	2.393 ± 0.151
AREB1	1.821 ± 0.275	2.291 ± 1.267	4.372 ± 0.255
ABF3	1.696 ± 0.269	2.086 ± 0.364	2.397 ± 0.316
DREB2A	2.256 ± 0.606	2.061 ± 0.773	0.767 ± 0.233
